# Impulse oscillometry identifies peripheral airway dysfunction in children with adenosine deaminase deficiency

**DOI:** 10.1186/s13023-015-0365-z

**Published:** 2015-12-18

**Authors:** Hirsh D. Komarow, Robert Sokolic, Michael S. Hershfield, Donald B. Kohn, Michael Young, Dean D. Metcalfe, Fabio Candotti

**Affiliations:** Laboratory of Allergic Diseases, National Institute of Allergy and Infectious Diseases, National Institutes of Health, NIH/NIAID/LAD/Bldg. 10, Room 1C129A1, 10 Center Drive, Bethesda, MD 20892-1960 USA; National Human Genome Research Institute, National Institutes of Health, Bethesda, MD 20892 USA; Department of Biochemistry, Duke University School of Medicine, Durham, NC USA; Department of Microbiology, Immunology and Molecular Genetics, University of California Los Angeles, David Geffen School of Medicine, Los Angeles, CA USA; Clinical Research Directorate/Clinical Monitoring Research Program, Leidos Biomedical Research, Inc., Frederick National Laboratory for Clinical Research, Frederick, MD 21702 USA; Division of Immunology and Allergy, University Hospital of Lausanne, CH-1101 Lausanne, Switzerland

**Keywords:** Adenosine deaminase deficiency, SCID, Children, Pulmonary dysfunction, Impulse oscillometry, Spirometry

## Abstract

Adenosine deaminase-deficient severe combined immunodeficiency (ADA-SCID) is characterized by impaired T-, B- and NK-cell function. Affected children, in addition to early onset of infections, manifest non-immunologic symptoms including pulmonary dysfunction likely attributable to elevated systemic adenosine levels. Lung disease assessment has primarily employed repetitive radiography and effort-dependent functional studies. Through impulse oscillometry (IOS), which is effort-independent, we prospectively obtained objective measures of lung dysfunction in 10 children with ADA-SCID. These results support the use of IOS in the identification and monitoring of lung function abnormalities in children with primary immunodeficiencies.

**Electronic supplementary material:**

The online version of this article (doi:10.1186/s13023-015-0365-z) contains supplementary material, which is available to authorized users.

## Background

Children with primary immunodeficiency diseases (PIDs) often suffer from severe and life-threatening illnesses. Adenosine deaminase (ADA) deficiency is among the most severe forms of PIDs, leading to severe combined immunodeficiency (SCID) and susceptibility to severe and recurrent opportunistic infections. In addition, the ubiquitous expression of ADA confers additional clinical phenotypes to patients affected with ADA-SCID that include non-infectious abnormalities of the lung that are incompletely characterized [1, 2]. Assessment of lung disease in children may employ X-ray and conventional tomography imaging, while lung function is usually evaluated using spirometry, which is effort-dependent and thus difficult to perform in younger patients. Impulse oscillometry (IOS) has been suggested as an alternative technique to assess lung function with a particular application to younger children and others unable to perform spirometry [3–5]. IOS is best understood as a technique that generates small pressure oscillations that are applied at the mouth and transmitted into the lungs, which in turn enables the measurement of resistance and reactance to the impedance of the respiratory system during spontaneous quiet breathing, and therefore provides an indirect quantification of lung function.

## Findings

### Subject characteristics

We assessed lung function in 10 children (3–18 years of age) with ADA-SCID by IOS and spirometry following informed consent. Seven patients were on treatment with PEG-ADA and 3 with gene therapy. Six patients had undergone CT imaging of their lungs and displayed: diffuse ground glass opacities (*n* = 3), parenchymal cysts (*n* = 2), mosaic attenuation (*n* = 4), bronchiectasis (*n* = 1), and nodules (*n* = 1). All patients could perform IOS while only 5 patients were able to complete spirometry (Table 1). In addition, 82 control subjects of ages 4–18 were evaluated following informed consent. Details regarding methodology, demographics, disease presentation, ADA activity, pulmonary and immune status are shown in Additional file 1: Table S1.

**Table 1:** Patient characteristics and pulmonary imaging and function testing

UPN	Age (years)	Gender	Ethnicity^a^	Treatment^b^	CT	Spirometry	IOS
1-ADA26	3	F	C	1	ND	-	+
2-ADA41	5	F	C	2	ND	-	+
3-ADA14	8	F	C	2	Normal	+	+
4-ADA16	9	M	H	1	dGGO, MA, B	-	+
5-ADA18	10	F	A	2	ND	+	+
6-ADA5	12	M	AA	1	MA	+	+
7-ADA46	14	F	C	2	dGGO, PC, MA, N	-	+
8-ADA17	16	F	AA	2	MA	-	+
9-ADA3	17	M	C	2	dGGO, PC	+	+
10-ADA47	18	M	AA	2	ND	+	+

^a^C = Caucasian, H = Hispanic, A = Asian, AA = African American

^b^1 = Gene Therapy; 2 = ADA conjugated with polyethylene glycol (PEG-ADA)

CT findings: *ND* CT not performed, *dGGO* diffuse ground glass opacities, *PC* parenchymal Cysts, *MA* Mosaic attenuation, *B* Bronchiectasis, *N* Nodules

+ Indicates successful completion of Spirometry and/or IOS

### Pulmonary function measurements

**Fig. 1:**
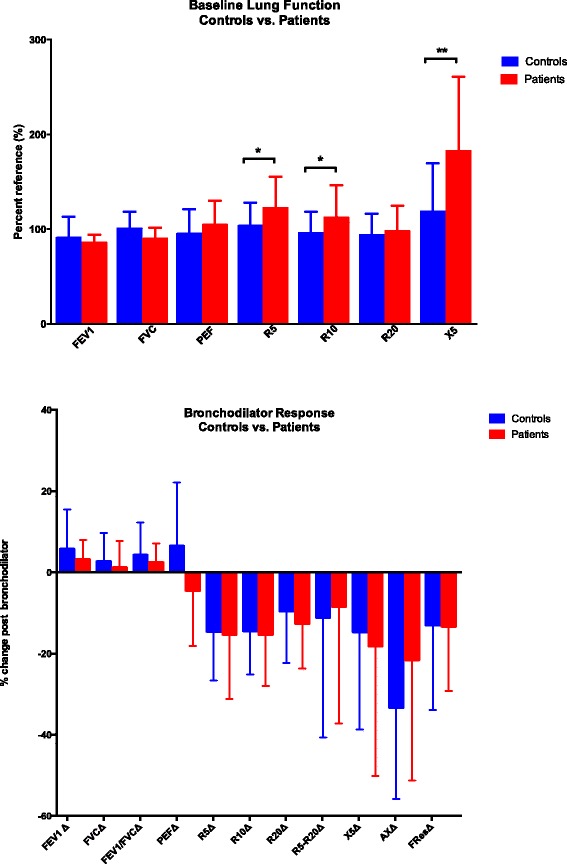
Baseline and Post bronchodilator Response. The mean % predicted values of all control subjects and patients with ADA-SCID as determined by spirometry and IOS is displayed at baseline (**a**) and the mean change of the bronchodilator response (**b**) with significance noted in baseline values for R5, R10 and X5 (p value; * < 0.05, ** <0.005)

Patient characteristics did not differ significantly when subdividing the entire cohort (*n* = 10) into patients that could (*n* = 5) and could not (*n* = 5) perform spirometry (due to intellectual or physical disability) and in comparison to age matched healthy controls (*n* = 82, Table 2). The mean baseline measurements of patients with ADA-SCID were within the normal range for spirometry and IOS (Table 3). With the exception of a higher expiratory peak flow (PEF), on average, ADA-SCID patients showed spirometry results similar to healthy controls (Table 3). IOS testing, however, revealed that the ADA-SCID patient cohort presented significant increases in baseline percent-predicted values for resistance at 5 Hz (R5, *p* = 0.032; Student *t*-test), and 10 Hz (R10, *p* = 0.044; Student *t*-test). Peripheral airway reactance was also significantly increased as indicated by higher X values at 5 Hz (X5, *p* = 0.001, data summarized in Fig. 1a) and change in X5 from reference (X5ref-X5, *p* = 0.041; Table 3, *italicized* values). Although, as a group, the R5-R20(%) of ADA-SCID patients did not differ significantly from control subjects, 4 patients had abnormal values (>35 %). Thus, patients with ADA-SCID displayed measurable defects in peripheral airway that was detected by IOS and not spirometry. A more detailed analysis focusing on individual patients revealed that 2 out of 5 patients who completed spirometry (based on FEV1) and 7 out of 10 patients who underwent IOS (based on R5, R5-R20% and X5ref-X5) had abnormal baseline pulmonary function (Additional file 1: Table S2).

**Table 2:** Pulmonary function testing in patients vs. controls

Characteristics	IOS (*n* = 10)	Spirometry able (*n* = 5)	IOS and not Spirometry (*n* = 5)	Controls (*n* = 82)	^a^ *P* value (*t*-test)
Age mean (SD)	11.2 (5.1)	13.0 (4.6)	9.4 (5.3)	9.22 (3.21)	0.090
Female Gender, n (%)	6 (60)	2 (40)	4 (80)	34 (41)	0.332
Height (cm)	135.9 (25.7)	146.3 (27.2)	125.5 (21.9)	136.22 (17.16)	0.957
Weight (kg)	44.3 (26.8)	54.6 (30.5)	33.9 (20.4)	39.64 (28.68)	0.630

^a^comparison of IOS (*n* = 10) to Controls

**Table 3:** Baseline results in patients vs. controls

Baseline Measurements						
	Mean (SD)		*P* value (*T*-test)	^a^Mean % Reference (SD)		*P* value (*t*-test)
Spirometry	Patients *n* = 5	Controls *n* = 82		Patients *n* = 5	Controls *n* = 82	
FEV1 (L)	2.2 (1.0)	1.85 (0.7)	0.354	86.0 (8.2)	90.8 (22.4)	0.636
FVC (L)	2.5 (1.2)	2.28 (0.9)	0.576	89.8 (11.8)	100.7 (17.6)	0.178
FEV1/FVC (%)	86.8 (3.4)	82.0 (9.9)	0.282			
PEF (L/sec)	6.2 (3.6)	4.0 (1.6)	*0.007*	104.8 (25.5)	95.1(25.8)	0.418
Impulse oscillometry	Patients *n* = 10	Controls *n* = 82		Patients *n* = 10	Controls *n* = 82	
R5 (cmH_2_0/L/sec)	8.4 (3.3)	7.8 (2.3)	0.488	122.3 (33.3)	103.4 (24.7)	*0.032*
R10 (cmH_2_0/L/sec)	6.9 (2.5)	6.4 (1.7)	0.451	112.3 (33.9)	95.9 (22.4)	*0.044*
R20 (cmH_2_0/L/sec)	5.7 (1.9)	5.4 (1.3)	0.592	98.0 (27.0)	93.8 (22.5)	0.592
R5-R20 (%)^Δ^	30.6 (9.4)	29.6 (11.0)	0.780			
X5 (cmH_2_0/L/sec)	−3.6 (1.8)	−3.0 (1.3)	0.231	182 (78.7)	118.7 (50)	*0.001*
AX (cmH_2_0/L)	27.5 (21.6)	22.4 (14.6)	0.323			
Fres Hz	22.2 (6.3)	20.5 (4.4)	0.280			
X5ref-X5 (cmH_2_0/L/sec)	1.3 (1.2)	0.35 (1.3)	*0.041*			

^a^Normal cut-off: Spirometry >80 % Reference; IOS: R5, R10, R20, <140 % Ref, X5ref-X5 < 1.5 cm H_2_0/L/sec, ^Δ^R5-R20 < 35 %

After bronchodilator administration, the mean response in ADA-SCID patients was within the normal range for both spirometry and IOS, except for an improved mean ΔR10 of −15.4 %, indicating borderline airways hyperreactivity (cutoff −15 %, Table 4, Fig. 1b). An individual analysis of the 4 patients that underwent post-bronchodilator spirometry revealed that none of the patients displayed reversibility (FEV1 cutoff, 12 % change). However, IOS testing was able to detect significant reversible obstruction in half of the cohort, including 2 of the 4 patients who did not show reversibility by spirometry (Additional file 1: Table S2).

**Table 4:** Bronchodilator response in patients vs. controls

Bronchodilator response^a^			
	Mean % change (SD)		*P* value (*T*-test)
Spirometry	Patients *n* = 4	Controls *n* = 82	
ΔFEV1	3.3 (4.7)	5.5 (9.7)	0.607
ΔFVC	1.3 (6.5)	2.7 (7.0)	0.341
ΔFEV1 / FVC	2.5 (4.6)	4.3 (8.1)	0.672
ΔPEF	−4.5 (13.7)	6.5 (15.5)	0.164
Impulse oscillometry	Patients *n* = 10	Controls *n* = 82	
ΔR5	−15.3 (15.9)	−14.6 (12.0)	0.438
ΔR10	−15.4 (12.5)	−14.5 (10.7)	0.809
ΔR20	−12.6 (11.1)	−9.6 (12.7)	0.483
ΔR5-R20	−8.5 (28.7)	−11.7 (29.8)	0.745
ΔX5	−18.2 (31.9)	−14.7 (24.0)	0.673
ΔAX	−21.6 (29.7)	−33.3(22.6)	0.139
ΔFres	−13.4 (15.8)	−13.1 (20.9)	0.961

^a^Airway reversibility was considered evident when there was an improvement in any of the following bronchodilator parameters : ΔR5 ≥ −20 %, ΔR10 ≥ −15 %, ΔR20 *≥ −*20 %, ΔAX *≥ −*45 %, and ΔFres *≥ −*25 %. By design, the percent improvement (reversibility) is displayed as a negative number because it indicates a magnitude decrease in resistance or reactance

Thus, in a cohort of 10 children with ADA-SCID, IOS was easily employed to assess dynamic lung function; while half of the patients could not complete spirometry testing. Baseline abnormality of pulmonary resistance (R) and reactance (X) was detected in the majority of ADA-SCID patients (70 %) using IOS. Undiagnosed reversible airway disease was revealed in half of the patients and only when using IOS. Also, in comparison to a pediatric control group of 82 patients, statistically significant abnormalities of peripheral airways were detected as indicated by measurement of airway resistance and reactance at lower frequencies (R5, R10, and X5).

## Discussion

Adenosine deaminase deficiency causes bronchial inflammation, pulmonary fibrosis and alveolar enlargements in *Ada* knockout mice [6–8]. Similarly, non-infectious lung abnormalities are emerging as frequent complications in patients with ADA-SCID. In prior reports, these abnormalities appeared to resolve upon enzyme replacement or transplantation [1, 2]. However, our results provide clinical evidence of continuing peripheral airway dysfunction in a significant fraction of patients receiving treatment resulting in sufficient correction of their immune function. These findings suggest that current therapeutic approaches such as ERT and gene therapy may be insufficient in preventing or controlling lung complications in ADA-SCID. Whether this is also the case of hematopoietic stem cell transplantation [9] remains to be investigated. Our IOS data indicates clinical evidence of continuing peripheral airway dysfunction in a significant fraction of patients that received treatment resulting in improvement of their immune function. The majority of patients with persistent lung disease (pneumonias, bronchiectasis) had abnormal findings on IOS. These observations appear to be independent of age and dAXP levels of diagnosis, and type of therapy employed (enzyme replacement or gene therapy). There were no correlations between the presence of lung abnormalities and demographics, therapeutic, and immunological parameters, however, we recognize that the small number of patients studied may have limited the ability to detect the effects of such variables. However, we believe it is important to caution care providers that ADA-SCID patients may benefit from therapies that target peripheral airway inflammation like inhaled corticosteroids and leukotriene inhibitors [10]. The long-term clinical significance in lung abnormalities in ADA-SCID patients is unknown, but our observations support the use of IOS for the identification and monitoring of this complication in children with this and other primary immunodeficiencies.

## Additional file

Additional file 1: **Online Supplementary Material.** **Table S1.** ADA-SCID patient cohort characteristics. **Table S2**. Baseline and Reversibility. (DOCX 75 kb)

## Abbreviations

ADA-SCID: Adenosine deaminase deficient severe combined immunodeficiency
IOS: Impulse Oscillometry
FVL: Flow-volume loop
PFTs: Pulmonary function tests
ERS/ATS: European respiratory society/American thoracic society
Z: Impedance
R: Resistance
X: Reactance
Hz: Hertz
