# Supplementary material for: Impulse oscillometry identifies peripheral airway dysfunction in children with adenosine deaminase deficiency
Source: Orphanet J Rare Dis. 2015 Dec 18;10:159. doi: 10.1186/s13023-015-0365-z (PMC4683718; doi:10.1186/s13023-015-0365-z)
Supplement: Supplementary file 1 — Online Supplementary Material. Table S1. ADA-SCID patient cohort characteristics. Table S2. Baseline and Reversibility. (DOCX 75 kb) [file 13023_2015_365_MOESM1_ESM.docx]

**Impulse oscillometry identifies peripheral airway dysfunction in children with adenosine deaminase deficiency**

**Additional File 1 for Online Supplementary Material**

**Methods**

*Subjects*

Ten children ages 3-18 years diagnosed with ADA-SCID were enrolled on NIH IRB approved protocol 00-HG-0209 following informed consent. All patients underwent a history, physical exam, and were evaluated prospectively with spirometry when possible, and IOS. Eighty-three control patients ages 4-18 were evaluated at the NIH pediatric clinic under protocol 05-I-0084 following informed consent. All control subjects were tested with spirometry and IOS and did not have clinical or laboratory findings suggestive of primary immunodeficiency or chronic systemic pulmonary or inflammatory diseases.

*Impulse Oscillometry*

The IOS system (MasterScreen Impulse Oscillometry by CareFusion, Yorba Linda, CA) was calibrated as suggested by the manufacturer. Testing and analysis was performed in accordance with ERS/ATS guidelines.([1](#_ENREF_1), [2](#_ENREF_2)) Briefly, subjects placed their lips around the mouthpiece of the IOS pneumotachometer and began breathing normally. A nasal clip was applied and the hands of the subject were placed on their cheeks to decrease their expansion during testing. Both lung resistance (R) and reactance (X), which reflect total pulmonary impedance (Z,) were measured and observed by the investigator in real-time, t(s), as a function of flow volume, (l), for 30-60 seconds. R, represents the energy required to propagate the pressure wave through the airways and X reflects the viscoelastic properties of the respiratory system. Values of reactance and resistance for frequencies of 5-20Hz were derived from each trial and stored. An average of 3 adequate trials of R and X values were analyzed and graphically displayed. If there was an indication in the real-time measurement of an abnormal tracing due to tongue or mouthpiece artifact, the trial was excluded. Children as from the age of 2 years can generally perform IOS with accurate and objective results. Predicted values for R and X were based on gender and height according to the equipment’s default normal references values as recommended by the manufacturer based on existing reference values. ([3](#_ENREF_3), [4](#_ENREF_4))

Cut off indices for normal/abnormal baseline values and reversibility were based on expert opinions in IOS and recent studies. ([2](#_ENREF_2), [5-9](#_ENREF_5)) Regarding baseline values, the following parameters were considered abnormal: for R5, R10, and R20 a percent reference of ≥140%, for R5-R20 a percent reference of >35%, and for X5ref-X5 a percent reference of ≥1.5 cm H_2_0/L/s. Airway reversibility was considered evident when there was an improvement in any of the following bronchodilator parameters: ΔR5 ≥-20%, ΔR10≥-15%, ΔR20≥-20%, ΔAX≥-45%, and ΔFres>-25%. By design, the percent improvement (reversibility) is displayed as a negative number because it indicates a magnitude decrease in resistance or reactance, thus for example a % change of R5 of -30% is a greater improvement in resistance than -20%.

Peripheral or small airway obstruction is reflected by increases in resistance, as seen in high R5-R20 and manifested in diseases such as asthma and chronic bronchitis. This is because the pressure wave signal propagating into the lung distal lung (R5) encounters greater resistance than the higher frequency more proximal (R20) impulse. Peripheral obstruction also results in a decrease in reactance because the signal returning from the lung periphery to the sensor also has to navigate these same narrowed airways, which will be reflected in lower X5, higher AX, and higher Fres. Proximal or upper airway obstruction leads to frequency-independent elevations in resistance and should have little to no effect on reactance, because the capacity of the lung to recoil and the signal to return to the sensor are relatively unaffected. Restrictive lung diseases will result in a decrease in reactance (X5), because the elastic recoil, and therefore the ability of the lung parenchyma to reflect the signal back, will be decreased. Restrictive processes should not affect resistance because the airway caliber is unchanged.

*Spirometry*

Flow-volume loop was determined by a Jaeger Masterscreen Spirometry system. The system was calibrated in accordance with ATS standards. All testing procedures were performed in accordance with ATS guidelines for determination of expiratory flow volume measurements. ([1](#_ENREF_1), [10](#_ENREF_10)) Forced expiratory volume (FEV_1_), forced vital capacity (FVC), FEV (25%-75%), and peak flow (PEF) were measured. Patients were instructed to exert maximal effort during forced expiratory and inspiratory maneuvers and the best of three acceptable recorded trials was reported. Percent predicted values were based on age, height, gender and ethnicity according to manufacturer’s standards. Cutoff for abnormal baseline values was <80% of predicted and airway reversibility was defined by an FEV1 % Δ of ≥ 12%.([11](#_ENREF_11))

One trained research nurse performed both spirometry and IOS measurements.

**Table S1: ADA-SCID patient cohort characteristics**

|  |  | **Age** | | | | **Mutation** | |  | **%dAXP** | | **Lung Infections** | | |  |
| --- | --- | --- | --- | --- | --- | --- | --- | --- | --- | --- | --- | --- | --- | --- |
| Subject no. | UPN | Presentation | Diagnosis (months) | Current management initiated | At testing (years) | *ADA* allele 1 | *ADA* allele 2 | ADA Activity (µmol/h/mL) | Diagnosis | At lung evaluation | Prior to Diagnosis | After diagnosis | During treatment | Neurologic Abnormalities |
| 1 | ADA26 | Birth | 0.8 | 1.5 y | 3.5 | c.646G>A (G216R) | c.646G>A (G216R) | 1.1 | 68.8 | 5.6 | None | Pneumonia | None | None |
| 2 | ADA41 | 5 mo | 42.8 | 19 mo | 5.2 | c.758G>C (R253P) | del c.955-959 | 0.2 | 17.3 | 0.3 | Pneumonias | None | None | None |
| 3 | ADA14 | 1 mo | 9.4 | 15 mo | 7.7 | c.478+1G>A | c.646G>A (G216R) | 0.0 | 14.8 | 0.2 | Pneumonias | None | None | Hearing impairment |
| 4 | ADA16 | 1 mo | 3.2 | 23 mo | 8.6 | c.424C>T (R142X) | c.976A>G (N326D) | 0.0 | 44.7 | 4.0 | Pneumonia | Pneumonia | Bronchiectasis | Hearing impairment |
| 5 | ADA18 | 10 wks | 2.6 | 3 mo | 9.5 | c.778G>A (E260K) | del exon 1 | 0.6 | 32.8 | 0.3 | Upper Respiratory infections | None | None | None |
| 6 | ADA5 | 7 mo | 7.1 | 8 y | 12.6 | not available | not available | 0.2 | 28.1 | 3.1 | Pneumonia | None | None | ADHD |
| 7 | ADA46 | 21 mo | 43.3 | 4 y | 13.9 | c.302G>A (R101Q) | c.646G>A (G216R) | 0.0 | 21.3 | 0.0 | Pneumonia | Pneumonia | Interstitial pneumonitis | None |
| 8 | ADA17 | 2 mo | 4.1 | 8.75 y | 15.8 | c.221G>T (G74V) | c.986C>T (A329V) | 6.3 | 3.3 | 0.3 | Pneumonias |  | Pneumonias | Cognitive delay; hearing impairment |
| 9 | ADA3 | 4 mo | 5.7 | 3 y | 17.2 | c.320T>C (L107P) | c.478+1G>A | 13.3 | 6.4 | 0.0 | Pneumonia |  | Pneumonias, bronchiectasis | Cognitive delay |
| 10 | ADA47 | 7 mo | 7.6 | 8 mo | 18.0 | not available | not available | 1.0 | 35.1 | 3.6 | Pneumonia | Pneumonia | Pneumonia | Cognitive delay |
|  |  |  |  |  |  |  |  |  |  |  |  |  |  |  |
|  |  |  |  |  |  |  |  | (n.v. 63.0±41.4) | (n.v. <0.2) | (n.v. <0.2) |  |  |  |  |

|  |  | **Immune Status** | | | | | | | | | | |
| --- | --- | --- | --- | --- | --- | --- | --- | --- | --- | --- | --- | --- |
| **Subject no.** | **UPN** | **Lymphocyte counts (cells/ul)** | **CD3 (cells/ul)** | **CD4 (cells/ul)** | **CD8 (cells/ul)** | **CD19 (cells/ul)** | **CD56 (cells/ul)** | **T Cell responses to PHA (Stimulation Index)** | **IgG (mg/dl)** | **IgA (mg/dl)** | **IgM (mg/dl)** | **IgE (IU/ml)** |
| 1 | ADA26 | 260 (n.v. 2300-5400) | 222 (n.v. 1400-3700) | 124 (n.v. 700-2200) | 75 (n.v. 490-1300) | 28 (n.v. 390-1400) | 10 (n.v. 130-720) | 566.8 | 749* (n.v. 441-1135) | 40 (n.v. 22-159) | 26 (n.v. 47-200) | 27.7 (n.v. 0.19-16.9) |
| 2 | ADA41 | 970 (n.v. 2300-5400) | 491 (n.v. 1400-3700) | 337 (n.v. 700-2200) | 110 (n.v. 490-1300) | 324 (n.v. 390-1400) | 156 (n.v. 130-720) | 602.5 | 952 (n.v. 463-1236) | 89 (n.v. 25-154) | 157 (n.v. 43-196) | 366 (n.v. 1.07-68.9) |
| 3 | ADA14 | 340  (n.v. 1900-3700) | 226 (n.v. 1200-2600) | 190 (n.v. 650-1500) | 24 (n.v. 370-1100) | 82 (n.v. 270-860) | 33 (n.v. 100-480) | 377.7 | 474* (n.v. 633-1280) | 8 (n.v. 33-202) | 6 (n.v. 48-207) | 10.7 (n.v. 1.03-161.3) |
| 4 | ADA16 | 1710 (n.v. 1900-3700) | 1110 (n.v. 1200-2600) | 441 (n.v. 650-1500) | 612 (n.v. 370-1100) | 46 (n.v. 270-860) | 549 (n.v. 100-480) | 941.2 | 1240 (n.v. 633-1280) | 53 (n.v. 33-202) | 112 (n.v. 48-207) | 612 (n.v. 1.03-161.3) |
| 5 | ADA18 | 360 (n.v. 1900-3700) | 120 (n.v. 1200-2600) | 55 (n.v. 650-1500) | 34 (n.v. 370-1100) | 53 (n.v. 270-860) | 196 (n.v. 100-480) | 97.8 | 1560 (n.v. 608-1572) | 95 (n.v. 45-236) | 186 (n.v. 52-242) | 798 (n.v. 0.98-570.6) |
| 6 | ADA5 | 190 (n.v. 1400-3400) | 163 (n.v. 1000-2200) | 91 (n.v. 530-1300) | 42 (n.v. 330-920) | 11 (n.v. 100-570) | 17 (n.v. 70-480) | 705.3 | 1028* (n.v. 639-1349) | 45 (n.v. 70-312) | 32 (n.v. 56-352) | 170 (n.v. 1.53-114) |
| 7 | ADA46 | 400 (n.v. 1400-3400) | 358 (n.v. 1000-2200) | 234 (n.v. 530-1300) | 88 (n.v. 330-920) | 16 (n.v. 100-570) | 23 (n.v. 70-480) | 250.8 | 1555* (n.v. 639-1349) | 667 (n.v. 70-312) | 160 (n.v. 56-352) | 513 (n.v. 1.53-114) |
| 8 | ADA17 | 2110 (n.v. 1400-3400) | 1891 (n.v. 1000-2200) | 595 (n.v. 530-1300) | 1247 (n.v. 330-920) | 152 (n.v. 100-570) | 72 (n.v. 70-480) | 924.1 | 2190* (n.v. 639-1349) | 203 (n.v. 70-312) | 62 (n.v. 56-352) | 2530 (n.v. 1.53-114) |
| 9 | ADA3 | 260 (n.v. 1400-3400) | 131 (n.v. 1000-2200) | 79 (n.v. 530-1300) | 44 (n.v. 330-920) | 53 (n.v. 100-570) | 75 (n.v. 70-480) | 133.7 | 1140* (n.v. 639-1349) | 102 (n.v. 70-312) | 45 (n.v. 56-352) | 839 (n.v. 1.53-114) |
| 10 | ADA47 | 320 (n.v. 1400-3400) | 234 (n.v. 1000-2200) | 125 (n.v. 530-1300) | 80 (n.v. 330-920) | 9 (n.v. 100-570) | 77 (n.v. 70-480) | 1263.2 | 944 (n.v. 639-1349) | 39 (n.v. 70-312) | 107 (n.v. 56-352) | 260 (n.v. 1.53-114) |
|  |  |  |  |  |  |  |  |  |  |  |  |  |
|  |  | Control values from: Shearer et al., JACI 2003, 112:973 | | | | | | (v.n. 249-4799) | Control values from: Jolliff et al., Clin Chem 1982; 28:126 * on Ig replacement therapy | | | |
|  |  |  |  |  |  |  |  |  |  |  |  |  |

**Table S2. Baseline and Reversibility**

| **Baseline** | | |  |  | |  | |  | |  | |  | |  | |  | |  | |  | |  |  |  |
| --- | --- | --- | --- | --- | --- | --- | --- | --- | --- | --- | --- | --- | --- | --- | --- | --- | --- | --- | --- | --- | --- | --- | --- | --- |
|  | **Spirometry** | | | | **IOS** | | | | | | | | | | | | | | | |  |  |  |  |
| **Patient** | **FEV1**  **(%Ref)** | **Normal Values (%)** | | | **R5**  **(%Ref)** | | **R10**  **(%ref)** | | **R20**  **(%ref)** | | **Normal Values (%)** | | **R5-R20**  **(%)** | | **Normal Values (%)** | | **X5ref-X5**  **(cm H_2_O/L/s)** | | **Normal Values**  **(cm H_2_O/L/s)** | |  |  |  |  |
| 1 | NA* |  | | | 104 | | 91 | | 85 | | <140 | | **40.7** | | <35 | | **1.98** | | <1.5 | |  |  |  |  |
| 2 | NA |  | | | 113 | | 102 | | 98 | | <140 | | **37.5** | | <35 | | **2** | | <1.5 | |  |  |  |  |
| 3 | 97 | >80 | | | 97 | | 95 | | 78 | | <140 | | **41.8** | | <35 | | 1.03 | | <1.5 | |  |  |  |  |
| 4 | NA |  | | | 134 | | 117 | | 92 | | <140 | | 31.2 | | <35 | | 1.05 | | <1.5 | |  |  |  |  |
| 5 | **79** | >80 | | | 123 | | 102 | | 98 | | <140 | | 24.1 | | <35 | | 0.59 | | <1.5 | |  |  |  |  |
| 6 | 87 | >80 | | | 104 | | 94 | | 72 | | <140 | | 26.5 | | <35 | | 1.23 | | <1.5 | |  |  |  |  |
| 7 | NA |  | | | 79 | | 71 | | 69 | | <140 | | 13.9 | | <35 | | -1.33 | | <1.5 | |  |  |  |  |
| 8 | NA |  | | | **201** | | **194** | | **160** | | <140 | | **40.8** | | <35 | | **3.32** | | <1.5 | |  |  |  |  |
| 9 | 90 | >80 | | | **141** | | 137 | | 120 | | <140 | | 22.3 | | <35 | | 1 | | <1.5 | |  |  |  |  |
| 10 | **77** | >80 | | | 127 | | 120 | | 108 | | <140 | | 27.6 | | <35 | | **1.77** | | <1.5 | |  |  |  |  |

| **Reversibility** | | |  |  | |  |  | |  | | |  | |  | | |  |  | |  |  |  |  |  |
| --- | --- | --- | --- | --- | --- | --- | --- | --- | --- | --- | --- | --- | --- | --- | --- | --- | --- | --- | --- | --- | --- | --- | --- | --- |
|  | **Spirometry** | | | | **IOS** | | | | | | | | | | | | | |  |  |  |  |  |  |
| **Patient** | **ΔFEV1**  **(%)** | **Reversible (%)** | | | **ΔR5**  **(%)** | **Reversible (%)** | **ΔR10**  **(%)** | **Reversible**  **(%)** | | **ΔR20**  **(%)** | **Reversible**  **(%)** | | **ΔAX**  **(%)** | | **Reversible (%)** | **ΔFres**  **(%)** | | **Reversible (%)** | **Number of**  **Positive for Reversibility** |  |  |  |  |  |
| 1 | NA |  | | | **-41** | >-20 | **-38** | >-15 | | **-38** | >-20 | | **-48** | | >-45 | **-46** | | >-25 | **5** |  |  |  |  |  |
| 2 | NA |  | | | -9 | >-20 | 2 | >-15 | | -2 | >-20 | | 30 | | >-45 | 7 | | >-25 | **0** |  |  |  |  |  |
| 3 | **10** | >12 | | | -9 | >-20 | -12 | >-15 | | -4 | >-20 | | -26 | | >-45 | -1 | | >-25 | **0** |  |  |  |  |  |
| 4 | NA |  | | | -7 | >-20 | **-16** | >-15 | | -11 | >-20 | | -13 | | >-45 | -10 | | >-25 | **1** |  |  |  |  |  |
| 5 | NA |  | | | 5 | >-20 | -2 | >-15 | | -2 | >-20 | | -5 | | >-45 | -22 | | >-25 | **0** |  |  |  |  |  |
| 6 | **0** | >12 | | | **-27** | >-20 | **-21** | >-15 | | -11 | >-20 | | -44 | | >-45 | -19 | | >-25 | **2** |  |  |  |  |  |
| 7 | NA |  | | | -7 | >-20 | -9 | >-15 | | -8 | >-20 | | 3 | | >-45 | -5 | | >-25 | **0** |  |  |  |  |  |
| 8 | NA |  | | | **-39** | >-20 | **-32** | >-15 | | **-24** | >-20 | | **-66** | | >-45 | -19 | | >-25 | **4** |  |  |  |  |  |
| 9 | **0** | >12 | | | **-20** | >-20 | **-18** | >-15 | | -13 | >-20 | | **-47** | | >-45 | -23 | | >-25 | **3** |  |  |  |  |  |
| 10 | **3** | >12 | | | 1 | >-20 | -8 | >-15 | | -13 | >-20 | | 0 | | >-45 | 4 | | >-25 | **0** |  |  |  |  |  |

*NA: not available (spirometry not completed, bronchodilator not administered); abnormal values in **bold.**

**References:**

1. Beydon N, Davis SD, Lombardi E, Allen JL, Arets HG, Aurora P, et al. An official American Thoracic Society/European Respiratory Society statement: pulmonary function testing in preschool children. Am J Respir Crit Care Med. 2007;175(12):1304-45.

2. Oostveen E, MacLeod D, Lorino H, Farre R, Hantos Z, Desager K, et al. The forced oscillation technique in clinical practice: methodology, recommendations and future developments. Eur Respir J. 2003;22(6):1026-41.

3. Dencker M, Malmberg LP, Valind S, Thorsson O, Karlsson MK, Pelkonen A, et al. Reference values for respiratory system impedance by using impulse oscillometry in children aged 2-11 years. Clinical physiology and functional imaging. 2006;26(4):247-50.

4. Nowowiejska B, Tomalak W, Radlinski J, Siergiejko G, Latawiec W, Kaczmarski M. Transient reference values for impulse oscillometry for children aged 3-18 years. Pediatr Pulmonol. 2008;43(12):1193-7.

5. Goldman MD, Saadeh C, Ross D. Clinical applications of forced oscillation to assess peripheral airway function. Respir Physiol Neurobiol. 2005;148(1-2):179-94.

6. H.J. Smith PR, M.D. Goldman. Forced oscillation technique and impulse oscillometry. Journals E, editor: ERS Journals LTD; 2005.

7. Marotta A, Klinnert MD, Price MR, Larsen GL, Liu AH. Impulse oscillometry provides an effective measure of lung dysfunction in 4-year-old children at risk for persistent asthma. J Allergy Clin Immunol. 2003;112(2):317-22.

8. Oppenheimer BW, Goldring RM, Herberg ME, Hofer IS, Reyfman PA, Liautaud S, et al. Distal airway function in symptomatic subjects with normal spirometry following World Trade Center dust exposure. Chest. 2007;132(4):1275-82.

9. Schermer T, Malbon W, Newbury W, Holton C, Smith M, Morgan M, et al. Spirometry and impulse oscillometry (IOS) for detection of respiratory abnormalities in metropolitan firefighters. Respirology. 2010;15(6):975-85.

10. Miller MR, Hankinson J, Brusasco V, Burgos F, Casaburi R, Coates A, et al. Standardisation of spirometry. Eur Respir J. 2005;26(2):319-38.

11. Reddel HK, Taylor DR, Bateman ED, Boulet LP, Boushey HA, Busse WW, et al. An official American Thoracic Society/European Respiratory Society statement: asthma control and exacerbations: standardizing endpoints for clinical asthma trials and clinical practice. Am J Respir Crit Care Med. 2009;180(1):59-99.
